# Clinical Challenges and Predictive Risk Factors for Outcomes in COVID-19–Associated Mucormycosis 

**DOI:** 10.30699/ijp.2025.2044252.3371

**Published:** 2025-07-01

**Authors:** Fardin Ahmadkhani, Seyed Jamal Hashemi, Roshanak Daei Ghazvini, Mohammadreza Salehi, Azin Tabari, Laura Alcazar-Fuoli, Farzad Pakdel, Alireza Abdollahi, Mohammadreza Firouzifar, Masoud Moradi, Sadegh Khodavaisy

**Affiliations:** 1 Department of Medical Parasitology and Mycology, School of Public Health, Tehran University of Medical Sciences, Tehran, Iran; 2 Research Center for Antibiotic Stewardship and Antimicrobial Resistance, Department of Infectious Diseases and Tropical Medicine, Imam Khomeini Hospital Complex, Tehran University of Medical Sciences, Tehran, Iran; 3 Otorhinolaryngology Research Center, Imam Khomeini Hospital Complex, Tehran University of Medical Sciences, Tehran, Iran; 4 Mycology Reference Laboratory, National Centre for Microbiology, Instituto de Salud Carlos III, Madrid, Spain; 5 Department of Oculo-Facial Plastic Surgery, Department of Ophthalmology, Farabi Hospital, Tehran University of Medical Sciences, Tehran, Iran; 6 Department of Pathology, Imam Khomeini Hospital Complex, Tehran University of Medical Sciences, Tehran, Iran; 7 Otolaryngology-Head and Neck Surgery, Otorhinolaryngology Research Center, Tehran University of Medical Sciences, Tehran, Iran; 8 Student Research Committee, Faculty of Health, Mazandaran University of Medical Sciences, Sari, Iran

**Keywords:** COVID-19, Mucormycosis, Risk Factors, Mortality, Diabetes Mellitus, Central Nervous System

## Abstract

**Background & Objective:**

Mucormycosis has emerged as a severe complication in COVID-19 patients, particularly among those with uncontrolled diabetes and those receiving corticosteroid therapy. The infection's tendency to spread from the sinuses to the orbit and central nervous system (CNS) significantly increases morbidity and mortality. This study aimed to identify clinical risk factors and outcomes associated with disease severity and mortality in COVID-19–associated mucormycosis (CAM), with a focus on disease progression to orbital and CNS involvement.

**Methods:**

A total of 180 confirmed CAM patients were enrolled and classified into three groups based on disease extent: sinus-only, sinus with orbital involvement, and sinus with both orbital and CNS involvement. Data were collected on demographics, clinical history, laboratory findings, imaging results, treatment modalities, and outcomes.

**Results:**

Of the 180 patients, 63.3% had sinus-only involvement, 23.9% had sinus and orbital involvement, and 12.8% had sinus, orbital, and CNS involvement. Uncontrolled diabetes was observed in 38% of patients and was more prevalent in those with extensive disease. Corticosteroid use was significantly associated with disease severity (*p* = 0.002). Invasive procedures, such as orbital exenteration, were significantly linked to CNS progression (*p* < 0.05). The overall mortality rate was 31% (55 of 180 patients).

**Conclusion:**

Uncontrolled diabetes and corticosteroid therapy are major risk factors for severe CAM. Extension of mucormycosis beyond the sinuses, particularly to the orbit and CNS, is associated with poor clinical outcomes and often requires aggressive surgical management. Early diagnosis and prompt intervention are essential to improve survival in these patients.

## Introduction

COVID-19–associated mucormycosis (CAM) has emerged as a formidable complication during the pandemic, contributing to significant morbidity and mortality in susceptible individuals. CAM is particularly prevalent among patients with uncontrolled diabetes mellitus and those treated with corticosteroids or other immunosuppressive agents (1). The pathogenesis of mucormycosis involves inhalation of fungal spores, which germinate within the paranasal sinuses, leading to rhino-orbital-cerebral mucormycosis (ROCM) (2). This form of infection is notably aggressive, especially when it invades critical anatomical structures such as the orbit and central nervous system (CNS), resulting in markedly high mortality rates (3, 4).

The progression of mucormycosis from the sinuses to the orbit and CNS is a complex process often mediated by angioinvasion, which enables the fungi to penetrate blood vessel walls, leading to tissue necrosis and hematogenous dissemination (3, 5, 6). The interaction between SARS-CoV-2 and mucormycosis is multifactorial. COVID-19–induced immune dysregulation, including lymphopenia and cytokine storm, impairs host defenses, while treatments such as corticosteroids and oxygen therapy further increase susceptibility to fungal infections. In diabetic patients, hyperglycemia impairs neutrophil function and promotes a favorable environment for fungal proliferation. As the infection spreads, angioinvasion by Mucorales species facilitates rapid extension to adjacent structures, potentially resulting in orbital cellulitis, cavernous sinus thrombosis, and brain abscesses (7, 8). Additional risk factors, including prolonged hospitalization, mechanical ventilation, and concurrent infections, can further compromise immune function and facilitate fungal spread (5, 9).

One of the major clinical challenges in CAM management is its rapid progression and diagnostic complexity. Presentations vary depending on the extent of involvement and may include facial pain, nasal congestion, periorbital edema, proptosis, and neurological deficits (3, 4, 6). The high mortality associated with CNS involvement underscores the urgency of early diagnosis and aggressive intervention. Cross-sectional imaging, particularly CT and MRI, is critical in evaluating the extent of disease, while histopathology and fungal culture remain the diagnostic gold standards (10).

Understanding the clinical spectrum and outcomes of CAM is essential for improving patient care. This study aims to examine the epidemiology, clinical characteristics, and outcomes of CAM, with a particular focus on identifying risk factors associated with disease severity and mortality. By investigating the roles of uncontrolled diabetes, corticosteroid exposure, and ICU-level care, we aim to generate actionable insights to support early recognition and improved management. Additionally, the study evaluates the impact of therapeutic strategies, including surgical debridement and antifungal therapy, on survival outcomes. Addressing these critical gaps in clinical knowledge is vital to reducing the burden of CAM and enhancing outcomes amid the ongoing COVID-19 pandemic.

## Materials and Methods

### Study Design and Setting

This observational study was conducted at Imam Khomeini Hospital Complex, a tertiary referral public hospital in Tehran, Iran, during the peak of the mucormycosis epidemic that coincided with the second wave of the COVID-19 pandemic, between July 2021 and December 2023. The study team comprised medical mycologists and clinical specialists, including ophthalmologists, otolaryngologists, and radiologists. Written informed consent was obtained from all participants, alongside the completion of a structured study questionnaire.

### Ethics Approval and Consent to Participate

The study was approved by the Ethical Committee of Tehran University of Medical Sciences (Approval Code: IR.TUMS.SPH.REC.1401.034). To protect patient confidentiality, no identifying information was included in the study documentation. Written informed consent was obtained from each participant prior to inclusion.

### Patient Selection and Grouping

Patients eligible for inclusion were those diagnosed with mucormycosis following confirmed SARS-CoV-2 infection, identified through active surveillance by the hospital’s infectious disease and medical mycology departments. Diagnosis of mucormycosis was confirmed via histopathological examination and/or positive culture of biopsy specimens.

Patients were categorized into three groups based on the anatomical extent of disease involvement:

- **Group A**: Sinus and orbital involvement
- **Group B**: Sinus, orbital, and central nervous system (CNS) involvement
- **Group C**: Sinus-only involvement

Group allocation was determined by ophthalmologists and otolaryngologists based on clinical manifestations and radiological imaging findings (CT and/or MRI).

### Inclusion Criteria

Patients were included if they met the following conditions:

(1) confirmed SARS-CoV-2 infection by real-time polymerase chain reaction (RT-PCR); (2) confirmed mucormycosis diagnosis based on histopathological findings and/or fungal culture; (3) evidence of involvement of the paranasal sinuses, orbits, or CNS; (4) clinical signs and symptoms consistent with mucormycosis (e.g., facial pain, nasal congestion, orbital swelling, proptosis); (5) imaging findings suggestive of sinusitis, orbital cellulitis, or brain involvement; and (6) positive fungal culture or histopathological evidence of fungal invasion compatible with mucormycosis.

### Exclusion Criteria

Patients were excluded if mucormycosis was not associated with COVID-19 or if their medical records were incomplete or lacked sufficient data for analysis.

### Data Collection

Data were collected prospectively during hospitalization using a structured questionnaire, covering the following domains: (1) socio-demographic profile; (2) current hospitalization; and (3) prior hospital admissions. All members of the research team received standardized training for accurate and uniform data collection.

Collected information included demographic data (age, sex), clinical history (presence of diabetes, corticosteroid use, duration of COVID-19 symptoms), laboratory findings (blood glucose, inflammatory markers), radiological findings (CT and MRI results), and treatment information (antifungal therapy, surgical interventions). Outcome measures, including recovery status, complications, and mortality, were also documented.

### Statistical Analysis

Descriptive statistics were used to summarize the data: means and standard deviations for quantitative variables, and frequencies and percentages for qualitative variables. Univariate logistic regression was initially performed to identify variables with *P* < 0.20, which were then included in the multivariate logistic regression model to assess independent predictors. A *P* value of less than 0.05 was considered statistically significant. Analyses were conducted using SPSS software, version 26 (SPSS Inc., Chicago, IL, USA).

## Results

### Study Population

A total of 180 patients with confirmed COVID-19–associated mucormycosis (CAM) were evaluated. Based on disease extent, patients were categorized into three groups: Group C (sinus-only involvement) included 114 cases (63.3%), Group A (sinus plus orbital involvement) included 43 cases (23.9%), and Group B (sinus, orbital, plus CNS involvement) included 23 cases (12.8%). The median age of the patients was 55.1 years (range 13–85 years). There were 79 males (43.9%) and 101 females (56.1%). Demographic characteristics and group comparisons are summarized in Table 1.

**Table 1:** Demographic characteristics of participants in study groups

Variable	Group C (N=114) (%)	Group A (N=43) (%)	Group B (N=23) (%)	All (N=180) (%)
Gender				
Female	65 (57)	27 (62.8)	9 (39.1)	101 (56.1)
Male	49 (43)	16 (37.2)	14 (60.9)	79 (43.9)
90 Days Survival				
Not Survived	32 (28.1)	12 (27.9)	11 (47.8)	55 (30.6)
Survived	82 (71.9)	31 (72.1)	12 (52.2)	125 (69.4)
Involvement Side				
Left	60 (52.6)	19 (44.2)	16 (69.6)	95 (52.8)
Right	50 (43.9)	20 (46.5)	7 (30.4)	77 (42.8)
Bilateral	4 (3.5)	4 (9.3)	0 (0)	8 (4.4)
ICA^1^ or CS^2^				
Involved	16 (14)	15 (34.9)	12 (52.2)	43 (23.9)
Not-involved	71 (62.3)	25 (58.1)	11 (47.8)	107 (59.4)
COVID-19 Admission				
Yes	37 (53.2)	24 (55.8)	9 (39.1)	70 (38.9)
No	11 (9.6)	11 (25.6)	8 (34.8)	30 (16.7)
PPF^3^				
Involved	28 (24.6)	19 (44.2)	17 (73.9)	64 (35.6)
Not-involved	64 (56.1)	17 (39.5)	5 (21.7)	86 (47.8)
Age (Mean ± S.E)	55.25 ± 1.17	53.19 ± 2.12	58.30 ± 2.31	55.16 ± 0.94
BMI^4^ (Mean ± S.E)	27.01 ± 0.77	26.20 ± 0.85	26.68 ± 1.05	26.63 ± 0.49

**Abbreviation**: 1: Internal carotid artery; 2: Cavernous Sinus; 3: Pterygopalatine fossa;4: Body mass index

**Table 2:** Comparison of underlying disease and other disease records of case in study groups

Variable	Group C N (%)	Group A N (%)	Group B N (%)	*P*_ Value
Demographic information				
Exenteration	2 (1.8)	3 (7)	4 (17.4)	0.005
Diabetes mellitus	73 (70.9)	31 (77.5)	15 (75)	0.709
ICU admission in COVID-19	48 (42.1)	29 (67.4)	16 (69.6)	0.003
Oxygen therapy in COVID-19	49 (43)	30 (69.8)	16 (69.6)	0.003
Corticosteroid therapy	58 (50.9)	34 (73.9)	17 (79.1)	0.002
Underlying disease				
Diabetic retinopathy	9 (8.6)	3 (7.3)	2 (8.7)	1.000
Malignancy	2 (1.8)	1 (2.3)	2 (8.7)	0.157
Diabetic nephropathy	8 (9.8)	3 (8.3)	1 (5)	0.915
Diabetic neuropathy	1 (1.2)	0 (0)	0 (0)	1.000
Cardiovascular disease	18 (15.8)	3 (7)	3 (13)	0.350
Hypertension	32 (29.4)	16 (38.1)	6 (26.1)	0.500
Cancer	4 (4.9)	1 (2.8)	2 (10)	0.404
Chemotherapy	5 (6)	2 (6.1)	1 (5)	1.000
Clinical manifestations of mucormycosis				
Ptosis	31 (27.2)	22 (51.2)	12 (52.2)	0.005
Proptosis	31 (27.2)	21 (48.8)	14 (60.9)	0.002
Chemosis	20 (17.5)	15 (34.9)	15 (65.2)	<0.001
Headache	44 (38.6)	30 (69.8)	12 (52.2)	0.002
Numbness	58 (50.9)	24 (55.8)	17 (73.9)	0.128
Eye’s pain	40 (35.1)	24 (55.8)	15 (65.2)	0.006
Ulcers	10 (9.1)	4 (9.3)	5 (21.7)	0.231
Nasal discharge	14 (12.6)	4 (9.3)	3 (13.6)	0.822
Necrosis	19 (16.7)	12 (27.9)	9 (39.1)	0.036
Decreased vision	56 (49.1)	32 (74.4)	18 (78.3)	0.002
Neurological disorders	10 (8.9)	7 (16.3)	3 (13)	0.360
Frozen eye	6 (5.3)	19 (44.2)	12 (52.2)	<0.001
Radiological Findings				
Sinus involvement	87 (92.6)	42 (97.7)	23 (100)	0.381
Orbital involvement	1 (1.1)	43 (100)	23 (100)	<0.001
CNS involvement	1 (1.1)	0 (0)	23 (100)	<0.001
Sinus thrombosis	0 (0)	10 (23.3)	20 (87)	<0.001
Vital signs^*^				
SPO2	95	95	96	0.011
Pulse rate	82.5	82	83	0.694
Respiratory rate	20	18	20	0.020
Systolic blood pressure	120	120	120	0.632
Diastolic blood pressure	75	80	72	0.373
Temperature	37	37	37	0.010

* The values ​​of the variables in this section are based on the median (IQR) calculation.

### Disease Progression

Radiological evaluation revealed that most patients had left-sided facial involvement. Pansinusitis was the most common pattern, followed by involvement of the ethmoid sinus (145 patients, 80.5%), maxillary sinus (134 patients, 74.4%), and sphenoid sinus (92 patients, 51.1%). In Group A, ethmoid, maxillary, and sphenoid sinus involvement occurred in 72%, 60%, and 51% of patients, respectively. These values increased to 95.6%, 70%, and 70%, respectively, in Group B. In contrast, Group C showed involvement in 53.5%, 56.1%, and 31.5% of patients, respectively.

Progression of infection from the sinuses to the orbit and CNS was significantly associated with severe clinical features, including proptosis (60.9%, *p* = 0.002) and frozen eye (52.2%, *p* < 0.001). Neurological deficits were also more prominent in Group B. Imaging findings demonstrated that sinus thrombosis was present in 87% of CNS-involved patients, with a statistically significant association (*p* < 0.001). Table 3 compares radiological and clinical progression between groups.

**Table 3:** Investigating the risk factors affecting the severity of the disease using logistic regression

Variable	Group A		Group B	
	OR (95 % CI)	P-Value	OR (95 % CI)	P-Value
SPO2	1.13(0.94, 1.36)	0.185	1.48(0.96, 2.26)	0.074
Respiratory rate	0.85(0.69, 1.04)	0.117	0.96(0.86, 1.08)	0.519
Systolic blood pressure	1.03(0.98, 1.07)	0.251	1.01(0.95, 1.07)	0.768
Diastolic blood pressure	1.01(0.94, 1.09)	0.726	0.99(0.89, 1.11)	0.903
Temperature	0.64(0.18, 2.24)	0.483	0.21(0.02, 2.44)	0.212
Hemoglobin	1.35(1, 1.81)	0.048	1.17(0.7, 1.94)	0.548
Sex	2.91(0.84, 10.15)	0.093	0.39(0.06, 2.59)	0.327
ICU admission	1.9(0.06, 59.96)	0.715	4.99(0.03, 718.52)	0.526
Oxygen therapy in COVID-19	6.6(0.14, 302.69)	0.334	9.99(0.08, 1236.8)	0.349
Facial numbness	1.44(0.38, 5.49)	0.589	3.51(0.32, 39.07)	0.307
Ptosis	2.35(0.68, 8.09)	0.176	0.37(0.05, 2.74)	0.329
Proptosis	0.82(0.19, 3.58)	0.796	0.67(0.06, 7.32)	0.745
Chemosis	0.84(0.17, 4.13)	0.829	2.59(0.38, 17.7)	0.333
Headache	3.37(0.81, 14.07)	0.095	0.78(0.09, 6.4)	0.813
Eye's pain	1.53(0.41, 5.69)	0.528	1.34(0.14, 13.13)	0.799
Necrosis of palate or nose	0.47(0.1, 2.31)	0.352	0.5(0.04, 6.95)	0.608
Decreased vision	2.89(0.77, 10.81)	0.115	4.53(0.48, 42.6)	0.187
Frozen eye	110.45(12.71, 959.69)	<0.001	24.05(1.51, 382.87)	0.024
History of corticosteroid use	0.3(0.03, 3.27)	0.323	0.02(0, 1.96)	0.096
Exenteration	2.05(0.1, 41.24)	0.640	192.56(4.04, 9184.16)	0.008
PPF	0.44(0.1, 1.95)	0.277	7.78(0.87, 69.9)	0.067
ICA or Cavernous sinus	0.64(0.15, 2.76)	0.546	3.9(0.58, 26.07)	0.160
Hospitalization due to COVID19	2.76(0.67, 11.44)	0.161	0.15(0.02, 1.5)	0.107
Malignancy	0.54(0.01, 51.81)	0.792	15.62(0.35, 695.78)	0.156
Cardiovascular diseases	0.1(0.02, 0.66)	0.017	0.07(0.01, 0.75)	0.028
Maxillary sinus	0.85(0.23, 3.16)	0.804	0.15(0.02, 1.3)	0.085
Sphenoid sinus	1.76(0.39, 8.03)	0.466	0.71(0.09, 5.61)	0.744
Sinus Debridement	0.74(0.19, 2.94)	0.666	11.94(0.36, 397.61)	0.166
Posaconazole	1.01(0.25, 4.16)	0.989	22.62(1.55, 329.83)	0.023
Complete systemic Amphotericin B	2.94(0.75, 11.56)	0.122	2.33(0.23, 23.85)	0.476
Orbital Injection	0.27(0.07, 0.97)	0.046	0.93(0.11, 7.57)	0.946

### Treatment and Outcomes

A total of 97 patients (54%) were treated with liposomal amphotericin B, and 64 patients (35%) received oral posaconazole as an adjunct to systemic therapy. Invasive procedures, including local orbital amphotericin B injection and surgical debridement, were utilized when indicated.

Use of oral posaconazole, frozen eye, and orbital exenteration were strongly predictive of progression to CNS involvement. The odds of being in Group B compared to Group C increased approximately 22-fold for posaconazole use, 24-fold for frozen eye, and 192-fold for orbital exenteration.

In terms of surgical interventions, 100 patients (55.5%) underwent endoscopic sinus surgery, 43 patients (21.2%) underwent orbital surgery, and 29 patients (6.1%) underwent brain surgery. The highest frequency of surgical procedures occurred in Group B (78.3%), followed by Group A (69.7%) and Group C (45.6%).

**Fig 1:**
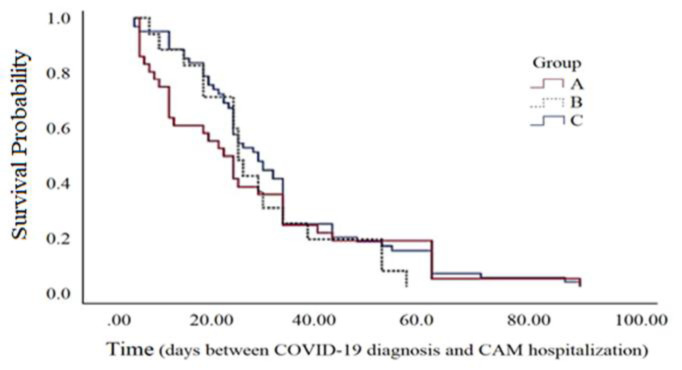
Kaplan–Meier curves of time interval (days) between getting COVID-19 and CAM.

Overall, 70 patients (38.9%) had been previously hospitalized due to COVID-19 before developing mucormycosis. Figure 1 shows the number of days it took for 50% of patients in each group to be hospitalized due to mucormycosis following COVID-19 diagnosis. Survival analysis comparing the time interval between COVID-19 infection and mucormycosis-related hospitalization across the three groups showed no statistically significant differences (Table 4).

**Table 4:** Median time (days) interval between getting COVID19 and Mucormycosis

P_Value	95% Confidence Interval	Median Time (S.E)	Group
0.582	(18.31 – 29.69)	24 (2.91)	C
	(12.21 – 23.80)	18 (2.96)	A
	(18.31 – 23.69)	21 (1.37)	B
	(18.28 – 23.73)	21 (1.39)	D

## Discussion

COVID-19–associated mucormycosis (CAM) has emerged as a significant complication during the pandemic, particularly among individuals with predisposing conditions such as uncontrolled diabetes mellitus and those treated with corticosteroids. The invasive nature of mucormycosis, particularly its progression from the paranasal sinuses to the orbital region and central nervous system (CNS), is associated with rapid clinical deterioration and high mortality.

The median age of CAM patients in our study was 55.1 years, consistent with findings from multiple studies. Garg et al. reported a median age of 50 years, and Muthu et al. found a similar median age of 53 years among CAM patients in India. An Iranian study also reported a median age of 51.7 years. Although these findings consistently point to a middle-aged to elderly demographic, Singh et al. reported a younger median age of 38 years, suggesting that CAM may also impact younger populations, possibly due to regional variations in underlying risk profiles or COVID-19 severity.

In contrast to the male predominance seen in many studies—including Toppo et al. and a multicenter investigation across 18 countries showing over 70% male prevalence—our study observed a slight female predominance (56.1%). While the gender distribution varies across studies, there is currently no definitive explanation for gender-related susceptibility to CAM, though differences in comorbidities or healthcare access may play a role.

The interval between COVID-19 diagnosis and mucormycosis hospitalization was not significantly different among patient groups in our study, echoing findings from Yadav et al. and Singh et al., who also observed a relatively uniform interval regardless of anatomical site of involvement. This suggests a rapid and consistent post-COVID onset of CAM symptoms across various presentations.

Diabetes mellitus—particularly uncontrolled diabetes—was a dominant risk factor in our cohort, found in 66% of all patients, with even higher prevalence among those with orbital (72%) and CNS involvement (75%). These findings are comparable with those from India, where diabetes was present in 73%–81% of CAM cases. In contrast, De Oruc et al. found no statistically significant association between diabetes and CAM severity, highlighting potential geographic or population-based differences. Our study reported a mean fasting blood glucose level of 170 mg/dL in patients with orbital involvement and HbA1c levels > 7.0% in 38% of all cases. Similar studies have documented HbA1c levels exceeding 10% in severe CAM, emphasizing the critical need for glycemic control.

The role of corticosteroid therapy in the development and progression of CAM was clearly evident, with usage in over 70% of Group B patients. This trend aligns with prior research, including studies by Garg et al. and CDC guidelines, which identify corticosteroids as a primary modifiable risk factor for CAM. The extensive use of corticosteroids in our study, including among patients with mild COVID-19, underscores the importance of judicious prescribing practices.

ICU admission and oxygen therapy were also strongly associated with CAM severity. Patients requiring ICU care or oxygen supplementation were more likely to experience orbital or CNS spread. These findings are supported by Patel, Mehta, and Chander et al., who linked prolonged hospitalization, mechanical ventilation, and high-flow oxygen with severe CAM manifestations. These interventions may compromise mucosal barriers and normal microbiota, facilitating fungal invasion.

Vital sign abnormalities—particularly in SpO₂, respiratory rate, and temperature—were more common in Groups A and B, indicating greater systemic involvement in severe CAM. This mirrors findings from other Iranian studies, reinforcing the prognostic value of these parameters.

The progression of rhino-orbital-cerebral mucormycosis (ROCM), facilitated by angioinvasion, leads to necrosis and rapid extension into the orbit and brain. Our radiological data support this, with sinus involvement observed in 92.6% of cases, orbital involvement in 23.9%, and CNS involvement in 12.8%. These values align with existing literature, which places CNS involvement between 7% and 24%. The pterygopalatine fossa (PPF) was involved in 35% of cases, especially in Group B, further highlighting its potential role as a conduit for intracranial extension.

Common presenting symptoms such as vision loss, facial numbness, and headache were consistent with prior studies by Muthu et al. and John et al., who also noted increased prevalence of ptosis, proptosis, and eye pain in severe CAM. Imaging findings in our study were consistent with those of Sarkar et al., showing clear correlation between radiologic patterns and clinical severity.

Surgical intervention was most frequent in patients with CNS involvement (78.3%), followed by those with orbital (69.7%) and sinus-only involvement (45.6%). The prevalence of eye discharge (17.4%) in Group B also supports the association between disease spread and orbital symptoms.

Treatment outcomes were improved through multidisciplinary management, combining systemic antifungal therapy—primarily liposomal amphotericin B—with surgical debridement. Posaconazole was used adjunctively in 35% of cases. Surgical intervention, when combined with antifungal therapy, significantly improved survival, especially in early stages. These findings are in line with Muthu et al., who recommended early aggressive treatment for improved outcomes.

Despite comprehensive treatment, survival was lowest in Group B (52%), compared to Groups A and C (72%). A large multicenter study showed that 46% of patients survived with combined liposomal amphotericin B and surgery, closely matching our findings. This highlights the critical importance of timely diagnosis, glycemic control, and appropriate antifungal therapy.

Our study emphasizes the necessity of early risk stratification, particularly in COVID-19 patients with uncontrolled diabetes or those receiving corticosteroids, oxygen, or ICU care. A multidisciplinary approach, involving infectious disease specialists, mycologists, radiologists, and surgeons, is essential for optimizing care. Preventive strategies, such as routine blood glucose monitoring and rational corticosteroid use, can significantly reduce CAM risk.

This study has limitations. Its cross-sectional design limits the ability to infer causality, and being single-center may restrict generalizability. Future multicenter studies are warranted to confirm these findings and explore novel therapeutic options, including adjunctive therapies such as hyperbaric oxygen and combination antifungal regimens. The role of immunomodulators in modulating disease progression should also be further investigated.

## Conclusion

In conclusion, uncontrolled diabetes mellitus and corticosteroid therapy are key predictors of severe CAM. Extension of infection to the orbit and CNS is associated with poor prognosis and higher mortality, often necessitating invasive surgical intervention. Early diagnosis, tight glycemic control, rational corticosteroid use, and a multidisciplinary treatment approach are essential to improving patient outcomes. These findings contribute valuable insights toward the management of CAM in the context of the ongoing COVID-19 pandemic.
